# A Direct Feedback FVF LDO for High Precision FMCW Radar Sensors in 65-nm CMOS Technology

**DOI:** 10.3390/s22249672

**Published:** 2022-12-10

**Authors:** Jun-Hee Lee, Mun-Kyo Lee, Jung-Dong Park

**Affiliations:** 1 Division of Electronics and Electrical Engineering, Dongguk University, Seoul 04620, Republic of Korea; 2 Yongin Research Institute, Hanwha Systems, Yongin-si 17121, Republic of Korea

**Keywords:** CMOS, low-dropout regulator, flipped voltage follower, large loop gain, fast transient, high unity-gain bandwidth

## Abstract

A direct feedback flipped voltage follower (FVF) LDO for a high-precision frequency-modulated continuous-wave (FMCW) radar is presented. To minimize the effect of the power supply ripple on the FMCW radar sensor’s resolution, a folded cascode error amplifier (EA) was connected to the outer loop of the FVF to increase the open-loop gain. The direct feedback structure enhances the PSRR while minimizing the power supply ripple path and not compromising a transient response. The flipped voltage follower with a super source follower forms a fast feedback loop. The stability and parameter variation sensitivity of the multi-loop FVF LDO were analyzed through the state matrix decomposition. We implemented the FVF LDO in TSMC 65 nm CMOS technology. The fabricated FVF LDO supplied a maximum load current of 20 mA with a 1.2 V power supply. The proposed FVF LDO achieved a full-spectrum PSR with a low-frequency PSRR of 66 dB, unity-gain bandwidth of 469 MHz, and 20 ns transient settling time with a load current step from 1 mA to 20 mA.

## 1. Introduction

Starting from military equipment, the FMCW radar sensor has broadened its application to an autonomous vehicle, a 3D imaging system, and a weather forecast. At the same time, the power management has become an integral part of the FMCW transceiver. To ensure the spatial and range resolution of the FMCW radar sensor, the power management circuit must supply stable and isolated supply voltages to each sensitive block, such as the PLL, mixer, and ADC [1,2,3,4,5,6,7,8,9,10]. With sawtooth modulation with *T_m_* = 2 ms, the time delay (*τ*) and the beat frequency (*f_b_*) for the frequency-modulated received signal from a target at a distance of *R* is given as

(1) $$\tau =\frac{2R}{v_{c}}$$

(2) $$f_{b}{=f}_{tx}-{\;f}_{rx}{=K}_{f}\;\tau$$

With *K_f_* of 500 GHz/s and a target range of 180 m, the maximum beat frequency is 600 kHz. Thus, the LDO should reject the low-frequency ripple from the supply to prevent it from degrading the phase noise of the PLL, which is the frequency modulation signal source. Moreover, even the power supply ripple of the frequency higher than the ADC sampling frequency may fold into the ADC in-band. Hence, it is essential for the LDO to reject a wide range of the power supply ripple, especially at the low-frequency range. We noticed that the FMCW frequency hopping approach [11] required an LDO to respond rapidly to the transient load variation. This is because the current consumption of the PLL changes relatively rapidly with the frequency hopping.

In order to achieve a high PSR across a wide frequency range, various analog circuit techniques have been introduced. A feedforward ripple cancellation achieves a high PSR by combining a feedback and feedforward signal path [12,13,14,15,16]. A bandgap reference (BGR) recursive configuration [17] and an output-supplied voltage reference [18] have been proposed to reduce the effect of a non-ideal PSR of the bandgap reference. A multi-loop structure [19,20,21,22,23] has been introduced to boost the unity-gain bandwidth and the transient response in various configurations. The flipped voltage follower (FVF) LDO [24] has become one of the most popular analog LDO approaches for the last decade. The FVF LDO has a local feedback loop that reduces output resistance. In addition, an independent control voltage generator can provide an adequate control voltage for the control transistor. However, the transient time of the local feedback loop is relatively slow due to the large pass transistor, and the unity-gain bandwidth of the LDO has been limited. A tri-loop FVF LDO with buffered FVF was proposed to achieve full-spectrum PSR and fast response time in [25]. Although additional loops through a tri-input EA provided more loop gain, the resulting low-frequency PSR was not sufficiently improved. A dual-loop FVF LDO was reported to provide full-spectrum PSR with high low-frequency PSR in [26]. As the control voltage regulating loop was removed, it created another power supply ripple path through the inverting stage, which necessitated an auxiliary LDO.

In this paper, a direct feedback FVF LDO was proposed. By constructing an error amplifier (EA) that directly controls the FVF local loop, the FVF LDO can eliminate the power supply ripple path, resulting in a high PSRR without the need for additional components. A local FVF loop with a super source follower realizes a fast transient response with a unity-gain bandwidth of 469 MHz, and an outer loop incorporating folded cascode EA enhanced a low-frequency PSR to 66 dB. State matrix decomposition [27] was applied to analyze the stability and parameter sensitivity of a multi-loop FVF LDO. 

This paper is organized as follows. Section 2 introduces the proposed direct-feedback LDO. The PSRR and stability analysis of the FVF LDO was also presented. State matrix decomposition [27] was employed to analyze the stability and parameter sensitivity of the multi-loop FVF LDO. Section 3 shows the experimental result with a fabricated FVF LDO, and Section 4 follows with a conclusion.

## 2. Design of FVF LDO

**Figure 1:**
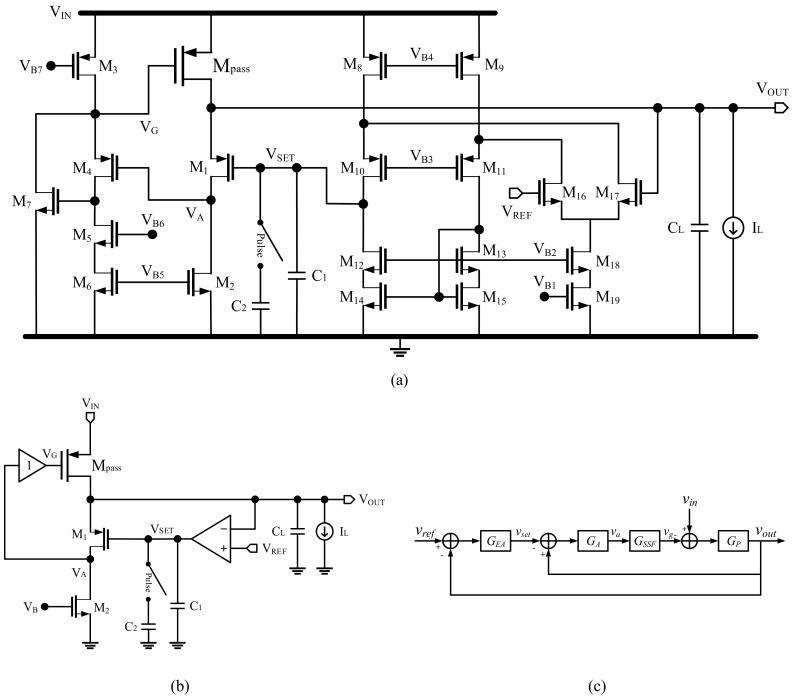
(**a**) Schematic diagram, (**b**) simplified schematic diagram, and (**c**) block of the proposed FVF LDO.

Figure 1 shows a schematic diagram of the proposed LDO regulator. The LDO consisted of a unity-gain buffer, an error amplifier (EA), an output capacitor, and transistors, M_pass_, M_1_, and M_2_. M_pass_, M_1_, and M_2_ formed a flipped voltage follower. Fast and weak shunt–shunt feedback loop 1 in the flipped voltage follower enables the fast response of the LDO. The output of the error amplifier, *V_SET_*, sets the input level of the flipped voltage follower. The input of the EA was connected to the reference input (*V_REF_*), and *V_OUT_* formed another feedback loop 2. This dramatically enhanced the open loop gain of the overall loop. Since *V_OUT_* was directly fed back into EA and the inverting stage was removed, we can eliminate the power supply ripple path without the need for an additional component. To enhance the transient performance, we needed to make the dominant pole of the fast loop 1 located at the output node. The output capacitor, *C*_L_, was connected to the output of the LDO to make the output node of the LDO dominant pole, and the capacitor, *C_1_*, was connected to the output of the error amplifier to stabilize loop 2. An additional compensation capacitor, *C_2_*, was enabled by a start-up pulse generator to guarantee more phase margin during the start-up situation. The unity-gain buffer was to drive the large power transistor, M_pass_. The size of the transistors, the capacitor values, and the load current (*I*_L_) values are listed in Table 1.

### 2.1. Fast Loop 1 Analysis

**Figure 2:**
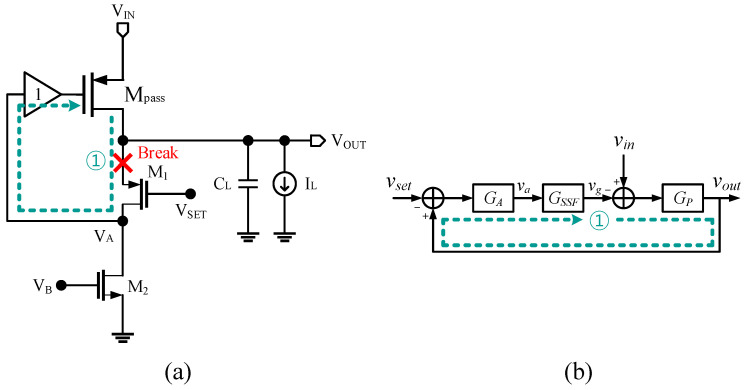
(**a**) FVF LDO without loop 2 and (**b**) its small-signal block diagram.

At higher frequencies where loop 2 did not work, only loop 1 worked. Without loop 2, the LDO simply had the flipped voltage follower (FVF) used as the power stage. The proposed LDO without loop 2 is shown in Figure 2a. The input *V_SET_* sets the output voltage of the FVF, and any interference or noise in the *V_IN_* works as a disturbance for the system. The series-shunt feedback structure reduced the output impedance of the system, enabling a high-frequency operation. The noise or interference from the power source was reduced by the internal feedback loop. To perform the PSRR analysis of the proposed LDO, we established a small-signal block diagram of the LDO. The block diagram is shown in Figure 2b. The *V_SET_* works as a reference input of the FVF, and any interference or noise in *V_IN_* was a disturbance for the system. The open-loop gain and output of LDO is

(3) $${LG}_{1}{=G}_{A}G_{SSF}G_{P}$$

(4) $$v_{out}=\;\frac{G_{A}G_{SSF}G_{P}}{{1+G}_{A}G_{SSF}G_{P}}v_{set}+\frac{G_{P}}{{1+G}_{A}G_{SSF}G_{P}}v_{in}\approx v_{set}+\frac{1}{G_{A}G_{SSF}}v_{in}$$

(5) $$G_{A}{=g}_{m1}\left(r_{o1}{||r}_{o2}\right)\frac{1}{1+s\left(r_{o1}{||r}_{o2}\right)C_{A}}$$

(6) $$G_{SSF\;}=\frac{K_{SSF}\;\omega_{n}^{2}}{s^{2}{+2\zeta \omega }_{n}{s+\omega }_{n}^{2}}$$

(7) $$G_{P}{=g}_{mP}\left(R_{L}{||r}_{oP}\right)\frac{1}{1+s\left(R_{L}{||r}_{oP}\right)C_{OUT}}$$

where *g_m_*_1_ is the transconductance of M_1_, *r_o_*_1_ and *r_o_*_2_ are the output resistance of M_1_ and M_2_, respectively, *C_A_* is capacitance seen at node A, *ω_n_* is the natural frequency of the super source follower, *ζ* is the damping factor of the super source follower, *g_mP_* is the transconductance of the pass transistor, *R_L_* is the load resistance, *r_oP_* is the output resistance of the pass transistor, and *C_OUT_* is the capacitance seen at the output node. Supply noise is reduced approximately by *G_A_* at high frequency. The bandwidth of the super source follower was boosted due to the internal feedback structure, and the pole at node A was also at high frequency, as M_1_ and M_2_ were small. The output capacitor, *C*_L_, was set such that the pass transistor, M_Pass_, was the slowest working component, and the dominant pole of the controller gain, *G_A_* and *G_SSF_*, were placed at a higher frequency. Therefore, loop 1 suppressed the supply noise through a wide frequency range. The supply noise at a higher frequency was absorbed by the large *C*_L_. The downside of loop 1 was that the open-loop gain was not large. Thus, the resulting PSRR of the LDO may not be sufficient only with loop 1. The error amplifier in loop 2 can improve the PSRR.

### 2.2. Slow Loop 2 Analysis

**Figure 3:**
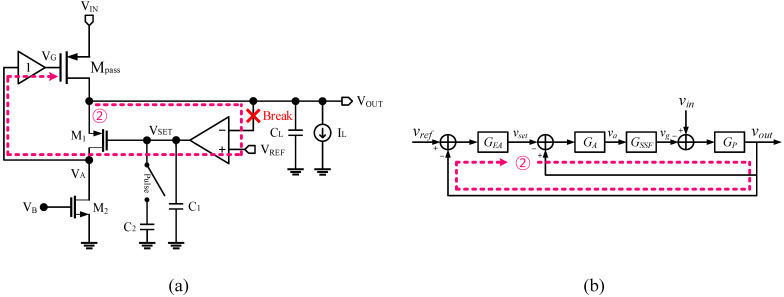
(**a**) Slow loop 2 broken at *V_SET_* and (**b**) its small-signal block diagram.

The folded cascode amplifier can drastically improve the closed-loop gain. Since *V_OUT_* was directly fed back into the EA and the inverting stage was removed, we could eliminate the power supply ripple path without the need for an additional component. Figure 3 shows the loop 2 feedback path. Breaking the loop at *V_SET_* gives

(8) $${LG}_{2}=\;G_{EA}\frac{G_{A}G_{SSF}G_{P}}{{1+G}_{A}G_{SSF}G_{P}}$$

(9) $$v_{out}=\;\frac{G_{EA}\frac{G_{A}G_{SSF}G_{P}}{{1+G}_{A}G_{SSF}G_{P}}}{{1+G}_{EA}\frac{G_{A}G_{SSF}G_{P}}{{1+G}_{A}G_{SSF}G_{P}}}v_{ref}+\frac{\frac{G_{A}G_{SSF}G_{P}}{{1+G}_{A}G_{SSF}G_{P}}}{{1+G}_{EA}\frac{G_{A}G_{SSF}G_{P}}{{1+G}_{A}G_{SSF}G_{P}}}\frac{1}{G_{A}G_{SSF}}v_{in}\;=\frac{G_{EA}G_{A}G_{SSF}G_{P}}{1+\left(1+G_{EA}\right)G_{A}G_{SSF}G_{P}}v_{ref}+\frac{G_{P}}{1+\left(1+G_{EA}\right)G_{A}G_{SSF}G_{P}}v_{in}\;\approx v_{ref}+\frac{1}{\left(1+G_{EA}\right)G_{A}G_{SSF}}v_{in}$$

(10) $$G_{EA}=\frac{K_{EA}}{\left(1+s/\omega_{p1}\right)\left(1+s/\omega_{p2}\right)}$$

where *G_EA_* is the voltage gain of the folded cascode amplifier. The PSRR is boosted approximately by *G_EA_*. Loop 1 is a unity-gain feedback network seen at node *V_SET_*, and the unity-gain bandwidth of loop 1 was far beyond that of the EA. Hence, we simply needed to compensate for the folded cascode EA. The folded cascode amplifier can be stabilized simply by adding the compensation capacitor, *C*_1_, to the output of the amplifier.

### 2.3. Overall Loop Analysis

**Figure 4:**
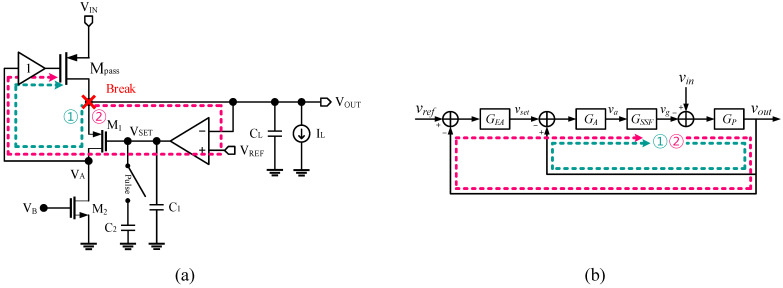
(**a**) Small-signal block diagram and (**b**) its simplified block diagram.

Loop 1 and Loop 2 formed a combined global loop. The global loop had the largest closed-loop gain, making it critical for the phase margin design. Figure 4 shows the combined diagram of loop 1 and loop 2. By breaking the loop at the node *V_G_*, the output voltage is expressed as

(11) $${LG=(1+G}_{EA})G_{A}G_{SSF}G_{P}$$

(12) $$v_{out}=\;\frac{\left(1+G_{EA}\right)G_{A}G_{SSF}G_{P}}{1+\left(1+G_{EA}\right)G_{A}G_{SSF}G_{P}}v_{ref}+\frac{G_{P}}{1+\left(1+G_{EA}\right)G_{A}G_{SSF}G_{P}}v_{in}\;\approx v_{ref}+\frac{1}{\left(1+G_{EA}\right)G_{A}G_{SSF}}v_{in}\;$$

**Figure 5:**
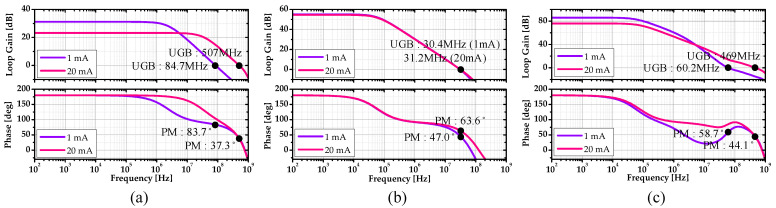
Phase margin simulation results of (**a**) Loop 1, (**b**) Loop 2, and (**c**) the overall loop.

Here, the open-loop gain had a dominant pole at the output of the EA, and the second pole was at the output of the LDO. The ${(1+G}_{EA})$ term in (11) made a quadratic zero near the unity-gain bandwidth of the EA. This zero was set to cancel out the second pole, which was below the unity-gain bandwidth of the LDO. It was noted that the LDO would be unstable without this zero. As a result, the ${(1+G}_{EA})$ term boosted the unity-gain bandwidth of the LDO. Figure 5 shows the phase margin simulation result. The unity-gain bandwidth of loop 1 was 507 MHz, and the phase margin was 37.3°. The unity-gain bandwidth of the loop 2 was 31.2 MHz, and the phase margin was 63.6°. The unity-gain bandwidth of the overall loop was 469 MHz, and the phase margin was 44.1°.

### 2.4. Effect of Non-Ideal PSRR of Each Component

**Figure 6:**
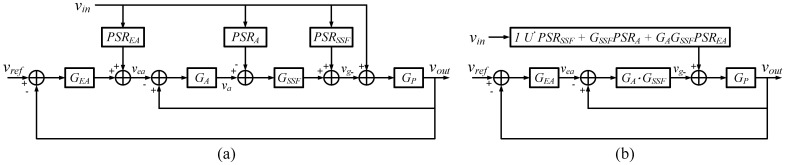
(**a**) Small-signal block diagram of the FVF LDO and (**b**) its simplified model.

There was more than one power supply ripple path in the FVF LDO. Circuit blocks with a non-ideal PSRR can provide an additional path for the power supply ripple. Figure 6 shows the effect of non-ideal components on PSRR. With the simplified model, the output of the LDO is given as

(13) $$v_{out}=\;\frac{\left(1+G_{EA}\right)G_{A}G_{SSF}G_{P}}{1+\left(1+G_{EA}\right)G_{A}G_{SSF}G_{P}}v_{ref}+\;\frac{G_{P}}{1+\left(1+G_{EA}\right)G_{A}G_{SSF}G_{P}}\left(1-{PSR}_{SSF}+G_{SSF}{PSR}_{A}+G_{A}G_{SSF}{PSR}_{EA}\right)v_{in}\;\approx v_{ref}+\frac{\alpha }{\left(1+G_{EA}\right)G_{A}G_{SSF}}v_{in}\;$$

where *PSR_SSF_* is the power supply rejection of the super source follower, *PSR_A_* is the power supply rejection of the FVF stage, and *PSR_EA_* is the power supply rejection of the folded cascode amplifier. The PSRR of the FVF stage and EA should be as low as possible. On the other hand, the super source follower with a poor PSRR helps the LDO reject the power supply ripple by working as a feedforward path.

### 2.5. Stability Analysis of Proposed LDO

Since the proposed LDO has two feedback loops, state matrix decomposition [27] must be more suitable for analyzing the stability than a classical open-loop ac analysis. Without looking at each loop separately, the closed-loop analysis gives a state space model as

(14) $$\left[\begin{array}{c} \dot{X_{1}}\\ \dot{X_{2}}\\ \dot{X_{3}}\\ \dot{X_{4}}\\ \dot{X_{5}}\\ \dot{X_{6}} \end{array}\right]=\left[\begin{array}{cccccc} 0 & 1 & 0 & 0 & 0 & 0\\ {-\omega }_{p1}\omega_{p2} & {-\omega }_{p1}{-\omega }_{p2} & 0 & 0 & 0 & {-K}_{P}\omega_{p1}\omega_{p2}\\ {-K}_{EA}\omega_{A} & 0 & {-\omega }_{A} & 0 & 0 & K_{P}\omega_{A}\\ 0 & 0 & 0 & 0 & 1 & 0\\ 0 & 0 & \omega_{n}^{2}K_{A} & {-\omega }_{n}^{2} & {-2\zeta \omega }_{n} & 0\\ 0 & 0 & 0 & {-K}_{SSF}\omega_{P} & 0 & {-\omega }_{P} \end{array}\right]\left[\begin{array}{c} X_{1}\\ X_{2}\\ X_{3}\\ X_{4}\\ X_{5}\\ X_{6} \end{array}\right]\;+\left[\begin{array}{cc} 0 & 0\\ 0 & \omega_{p1}\omega_{p2}\\ 0 & 0\\ 0 & 0\\ 0 & 0\\ \omega_{p}\left({1-PSR}_{SSF}{+K}_{SSF}{PSR}_{A}{+K}_{A}K_{SSF}{PSR}_{EA}\right) & 0 \end{array}\right]\left[\begin{array}{c} v_{in}\\ v_{ref} \end{array}\right]$$

(15) $$\left[\begin{array}{c} v_{set}\\ v_{a}\\ v_{g}\\ v_{out} \end{array}\right]=\left[\begin{array}{cccccc} K_{EA} & 0 & 0 & 0 & 0 & 0\\ 0 & 0 & K_{A} & 0 & 0 & 0\\ 0 & 0 & 0 & K_{SSF} & 0 & 0\\ 0 & 0 & 0 & 0 & 0 & K_{p} \end{array}\right]\left[\begin{array}{c} X_{1}\\ X_{2}\\ X_{3}\\ X_{4}\\ X_{5}\\ X_{6} \end{array}\right].$$

The detailed closed-loop analysis is shown in the Appendix A. The LDO is asymptotically stable when all the real parts of the eigenvalues of matrix A are negative. The eigenvalues are given as

(16) $$\lambda_{1}=-5.543\times {\;10}^{9}+j4.612\times {\;10}^{9}\;\lambda_{2}=-5.543\times {\;10}^{9}-j4.612\times {\;10}^{9}\;\lambda_{3}=-1.414\times {\;10}^{9}+j4.342\times {\;10}^{9}\;\lambda_{4}=-1.414\times {\;10}^{9}-j4.342\times {\;10}^{9}\;\lambda_{5}=-3.444\times {\;10}^{8}+j2.297\times {\;10}^{8}\;\lambda_{6}=-3.444\times {\;10}^{8}-j2.297\times {\;10}^{8}$$

**Figure 7:**
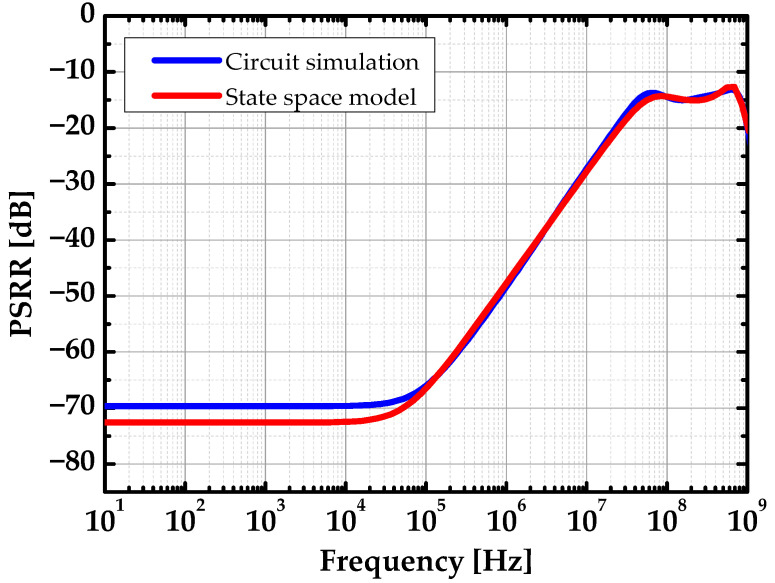
PSRR of the proposed LDO.

Since all the eigenvalues have negative real parts, the LDO was asymptotically stable. The parameters used in the analysis are given in Table 2. The parameters were extracted from the circuit simulation results, including parasitics. Figure 7 compares the PSRR simulation results from the circuit simulator and state space model. The state space model fits the circuit simulation result and can predict the pole/zero location of the transfer function.

The red line represents the simulation result with the state space model, and the blue line represents the simulation result with Cadence Spectre. We also identified the parameter variation sensitivity by computing the real part of the critical eigenvalue with variation in each parameter. Plotting the highest real part of the eigenvalues, the circuit should follow the conditions:

(17) ∀λi, Re(λi) < 0 

**Figure 8:**
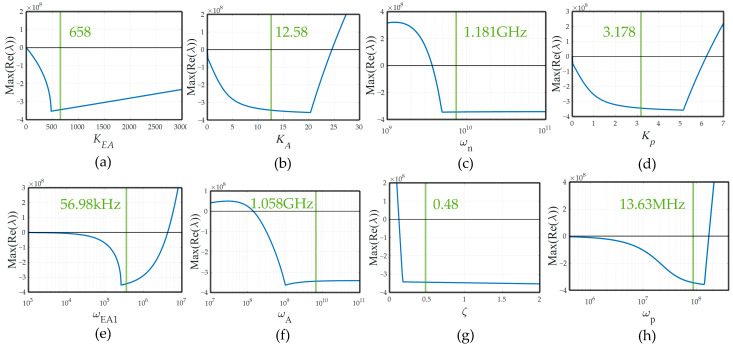
Parameter sensitivity simulation result for (**a**) the voltage gain of the folded cascode EA, (**b**) the voltage gain of the FVF stage, (**c**) the natural frequency of SSF, (**d**) the voltage gain of the pass transistor, (**e**) the dominant pole at the folded cascode EA, (**f**) the pole at the FVF stage, (**g**) the damping factor of SSF, (**h**) the pole at the output.

Figure 8 shows parameter variation sensitivity simulation results with various circuit parameters. Nominal design values are marked as the green line.

## 3. Measurement Results

**Figure 9:**
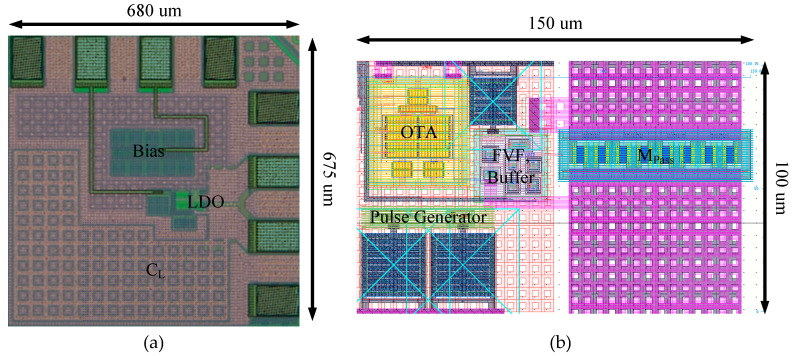
(**a**) Chip photograph of the fabricated FVF LDO and (**b**) layout of the FVF LDO.

We implemented the LDO in TSMC 65 nm CMOS technology with an active area of 0.037 mm^2^, including a 350 pF on-chip output capacitor. Figure 9 shows a chip photograph of a fabricated FVF LDO. A 350 pF output capacitor was implemented on-chip using a MOM capacitor. We performed the on-chip probe measurements and the chip-on-board measurements.

**Figure 10:**
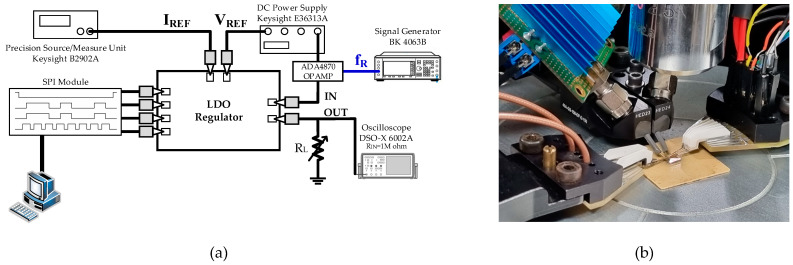
(**a**) Schematic diagram of the PSRR measurement setting and (**b**) a photograph of the measurement setting.

**Figure 11:**
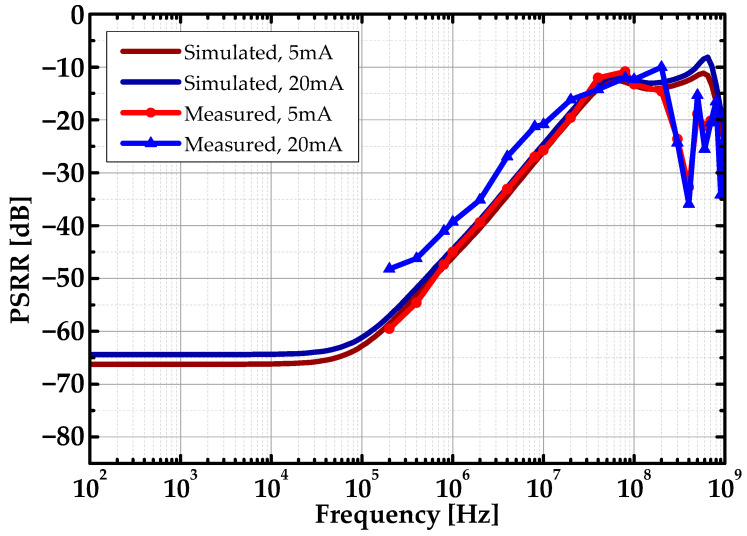
Simulated and measured PSRR of the FVF LDO.

The power supply rejection ratio measurement setting is shown in Figure 10. The Analog Device ADA4870 OPAMP supplied the DC power and ac ripple at the frequency of f_R_ to the LDO. The OPAMP was used to reduce the output impedance and combine the DC voltage with the ac ripple. A Keysight E36313A DC power supply sets the reference voltage and voltage bias for the OPAMP. A BK Precision BK4063B arbitrary signal generator provided the input ripple signal to the OPAMP. A Keysight B2902A SMU supplied I_ref_ to bias the internal amplifiers and buffer. The biasing point was controlled by the SPI Module. A Keysight DSO-X oscilloscope was used to measure the input and output ripple. The PSRR was calculated using measured input and output. Figure 11 shows the PSRR measurement result. The fabricated FVF LDO achieved a full-spectrum PSR of 64.6 dB at 100 kHz and the worst measured PSRR of 10 dB at 200 MHz. 

**Figure 12:**
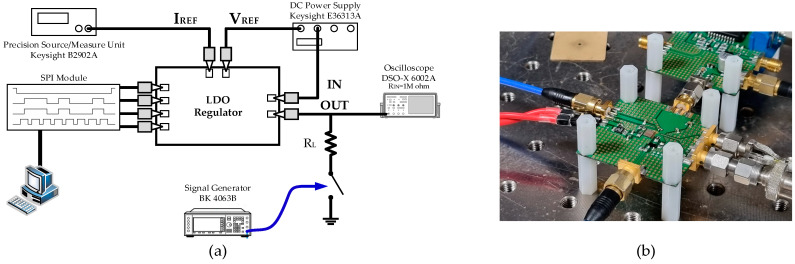
(**a**) Schematic diagram of the load transient measurement setting and (**b**) a photograph of the measurement setting.

**Figure 13:**
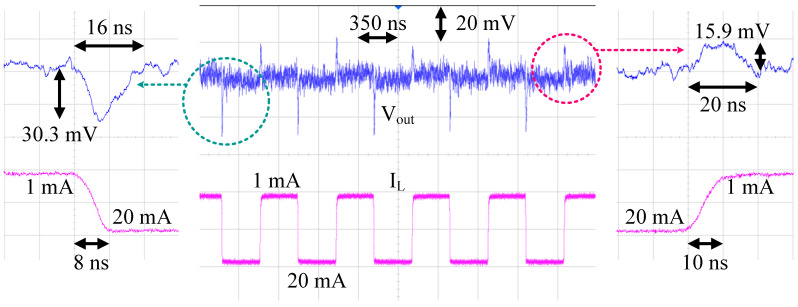
Load transient measurement result.

The load transient measurement setting is shown in Figure 12. A Keysight E36313A was used to supply *V_IN_* and *V_REF_* to the LDO, and a Keysight B2902A was used to input I_REF_ to bias the internal amplifiers and buffer. The load control signal was given from the BK precision BK4064B arbitrary signal generator. The load current was stepped from minimum to maximum, with an edge time of 8 ns. The load transient measurement result is given in Figure 13. The maximum voltage droop was 30.3 mV, and the settling time was about 16 ns. Transient load regulation was 141 µV/mA.

**Figure 14:**
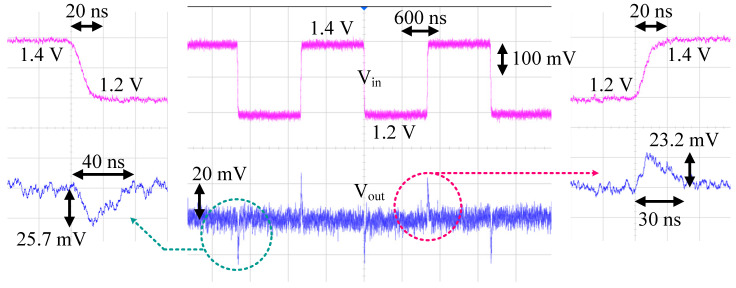
Line transient measurement result.

The line transient measurement setting was the same as the PSRR measurement setting, and the only difference was that the ripple signal, f_R_, was replaced with a square wave. The line transient measurement result is given in Figure 14. With the power supply voltage changing from 1.2 V to 1.4 V within 20 ns, the output voltage changed by about 25.7 mV. The settling time to the final value was about 40 ns.

Table 3 summarizes the performance of the proposed FVF LDO with other state-of-the-art LDOs. The proposed FVF LDO occupied a 0.037 mm^2^ active area. The LDO output was 1 VDC with a supply voltage of 1.2 VDC. The maximum output current was 20 mA, and the quiescent current was 290 µA. An output capacitor of 350 pF was used. The worst-case load transient overshoot was 30.3 mV with a load current step of 8 ns edge time, and the output was settled within 16 ns. When the response time of the LDO is comparable to the edge time, the assumption in the simple response time equation [28] is no longer valid. Assuming that the load current varies at a constant rate [29], the response time is given as

(18) $$T_{R}=\sqrt{\frac{{2C}_{L}{\Delta V}_{o}T_{\mathrm{edge}}}{{\Delta I}_{L}}}$$

The shorter the response time, the better the performance is. The response time, calculated according to (18), is shown in Table 2. The response time of the LDO was 2.99 ns. Transient FoM [28] is given by

(19) $${\mathrm{FoM}=T}_{R}\frac{I_{Q}}{I_{L(max)}}$$

where the smaller FoM represents better performance. The proposed FVF LDO achieved an FoM of 43.4 ps. The low-frequency PSRR of the FVF LDO was 66 dB, and the worst-measured PSRR of the LDO was 10 dB at 200 MHz.

## 4. Discussion

The proposed FVF LDO was successfully implemented in 65 nm CMOS technology. The PSRR measurement results confirmed that the analytic model and simulation results corresponded quite well with the measured PSRR. Our work has demonstrated that a simple direct feedback structure could improve low-frequency PSRR without additional components. The proposed LDO operated stably with various line/load transient situations, and the output settled rapidly to the final value. For future research, current efficiency can be improved by using an efficient buffer structure or an adaptive bias scheme.

## 5. Conclusions

A direct feedback flipped voltage follower (FVF) LDO was proposed. Both the classical ac analysis and the state-space model of the LDO were performed, and the results were compared with the circuit simulations. The parameter variation sensitivity of the LDO was also investigated using the state matrix model. The local FVF loop achieved a fast response and a high unity-gain frequency, and the outer loop with the folded cascode error amplifier (EA) enhanced the low-frequency closed-loop gain. The proposed direct feedback structure had a less power supply ripple path without a complex design. Experimental results verified theoretical predictions.

## Figures and Tables

**Table 1:** List of the component values in the proposed FVF LDO.

Component	Value	Component	Value
M_1_	8 µm/0.13 µm	M_8_, M_9_	60 µm/1 µm
M_2_	4 µm/0.13 µm	M_10_, M_11_	40 µm/1 µm
M_3_	14 µm/0.18 µm	M_12_, M_13_	12 µm/1 µm
M_4_	3 µm/0.06 µm	M_14_, M_15_	12 µm/1 µm
M_5_, M_6_	2 µm/0.18 µm	M_16_, M_17_	10 µm/1 µm
M_7_	3 µm/0.18 µm	M_18_, M_19_	12 µm/1 µm
*C*_L_	350 pF	*I*_L_	1 mA–20 mA

**Table 2:** Parameters used in the state space model.

Parameter	Value	Parameter	Value
$K_{EA}$	657.9	$K_{A}$	12.576
$\omega_{p1}$	2π × 5.698 × 10^4^	$\omega_{A}$	2π × 1.058 × 10^9^
$\omega_{p2}$	2π × 1.194 × 10^8^	${PSR}_{A}$	0.02778
${PSR}_{EA}$	0.05833	$K_{SSF}$	0.8386
PDF+1	3.178	$\omega_{n}$	2π × 1.181 × 10^9^
$\omega_{P}$	2π × 1.363 × 10^7^	ζ	0.4799
		${PSR}_{SSF}$	0.0104

**Table 3:** Performance comparison with state-of-the-art LDOs.

LDO Regulator	This Work	[12]	[26]	[29]	[30]
Type	Analog	Analog	Analog	Analog	Hybrid
Process (nm)	65	130	65	130	40
Area (mm^2^)	0.037	0.049	0.053	0.008	0.056
*V_in_* (V)	1.2	1.15	1.2	1–1.4	1.25–1.4
*V_out_* (V)	1	1	1	0.8	1.1–1.25
*I*_Q_ (µA)	290	50	27–82	112	300
Max. I_load_ (mA)	20	25	20	25	245
Load capacitor (nF)	0.35	4000	0.3	0.025	20
Load transient Overshoot (mV)	30.3 in ns step	15 in 10 ns step	71 in 0.8 ns step	48 in 3 ns step	71 in 0.3 µs step
*T*_R_ (ns)	2.99	438	5.35	0.881	73
Transient FoM (ps)	43.4	438	1.45	0.9	226
Settling Time @Max. current step (ns)	16	500 *	200	80	520
PSRR (dB)	66 at 1 kHz ^†^ 43.5 at 1 MHz 23.5 at 10 MHz	60 at 1 kHz 67 at 1 MHz	60 at 1 kHz 42 at 1 MHz 10 at 100 MHz	63 at 1 kHz 57 at 1 MHz 22 at 10 MHz	50 at 1 kHz 43 at 1 MHz 25 at 10 MHz
Load regulation (µV/mA)	141	48	15	173	24
Line Regulation (mV/V)	1.04	26	1	2.25	3.16

* Estimated from figure. ^†^ Simulated.

## Appendix A

Let $X_{1}{=v}_{set}/K_{EA}$ be a state variable, and the gain of the error amplifier is

(A1) GEA=vsetvref  − vout=KEA(1+sωp1)(1+sωp2).

Substituting $v_{set}{=K}_{EA}X_{1}$ into (A1) and identifying the numerator and the denominator,

(A2) vref − vout=X1+(1ωp1+1ωp2)X1˙+1ωp1ωp2X1¨.

Let a state variable $X_{2}=\dot{X_{1}}$, and when substituting it into (A2),

(A3) $$\dot{X_{2}\;}{=-\omega }_{p1}\omega_{p2}X_{1}\;-\;\left(\omega_{p1}{+\omega }_{p2}\right)X_{2}\;{-\;\omega }_{p1}\omega_{p2}v_{out}{+\omega }_{p1}\omega_{p2}v_{ref}.$$

Let $X_{3}{=v}_{a}/K_{A}$ be a state variable, and the gain of the error amplifier is

(A4) GA=vavref − vset=KA1+sωA.

Substituting $v_{a}{=K}_{A}X_{3}$ into (A4) and identifying the numerator and the denominator,

(A5) $$\dot{X_{3}\;}{=-K}_{EA}\omega_{A}X_{1}\;{-\;\omega }_{A}X_{3}{+\omega }_{A}v_{out}.$$

Let $X_{4}{=v}_{g}/K_{SSF}$ be a state variable, and the gain of the super source follower is

(A6) GSSF=vgva=KSSF1+2ζωns+1ωn2s2.

Substituting $v_{g}{=K}_{SSF}X_{4}$ into (A6) and identifying the numerator and the denominator,

(A7) va=KAX3=X4+2ζωnX4˙+1ωn2X4¨

Let a state variable $X_{5}=\dot{X_{4}}$, and when substituting it into (A7),

(A8) $$\dot{X_{5}}{=\omega }_{n}^{2}K_{A}X_{3}\;{\;-\;\omega }_{n}^{2}X_{4}\;{\;-\;2\zeta \omega }_{n}X_{5}.$$

Let $X_{6}{=v}_{out}/K_{P}$ be a state variable, and the gain of the pass transistor is

(A9) GP=voutvsgP=KP1+sωP.

Assuming the PSR of each component is constant, the effective source-gate voltage *v_sgP_* is

(A10) $$v_{\mathrm{sgP}}=\left({1\;-\;PSR}_{SSF}{+K}_{SSF}{PSR}_{A}{+K}_{A}K_{SSF}{PSR}_{EA}\right)v_{in}\;{-\;v}_{g}.$$

Substituting (A10), $v_{out}{=K}_{P}X_{6}$ and $v_{g}{=K}_{SSF}X_{4}$ into (A9), and identifying the numerator and the denominator,

(A11) $$\dot{X_{6}}{=-K}_{SSF}\omega_{P}X_{4}\;{-\;\omega }_{P}X_{6}{+\omega }_{P}\left({1\;-\;PSR}_{SSF}{+K}_{SSF}{PSR}_{A}{+K}_{A}K_{SSF}{PSR}_{EA}\right)v_{in}.$$

Substituting $v_{out}{=K}_{P}X_{6}$ into (A3) and (A5), we finally obtain

(A12) $$\{\begin{array}{l} \dot{X_{1}}{=X}_{2}\;\\ \dot{X_{2}}{=-\omega }_{p1}\omega_{p2}X_{1}\;-\;\left(\omega_{p1}{+\omega }_{p2}\right)X_{2}{\;-\;\omega }_{p1}\omega_{p2}K_{P}X_{6}{+\omega }_{p1}\omega_{p2}v_{ref}\\ \dot{X_{3}}{=-K}_{EA}\omega_{A}X_{1}-\omega_{A}X_{3}{+\omega }_{A}K_{P}X_{6}\\ \dot{X_{4}}{=X}_{5}\\ \dot{X_{5}}{=\omega }_{n}^{2}K_{A}X_{3}{\;-\;\omega }_{n}^{2}X_{4}{\;-\;2\zeta \omega }_{n}X_{5}\\ \dot{X_{6}}{=-K}_{SSF}\omega_{P}X_{4}{\;-\;\omega }_{P}X_{6}{+\omega }_{P}\left({1\;-\;PSR}_{SSF}{+K}_{SSF}{PSR}_{A}{+K}_{A}K_{SSF}{PSR}_{EA}\right)v_{in} \end{array}$$

(A13) $$\{\begin{array}{c} X_{1}{=v}_{set}/K_{EA}\\ X_{2}=\dot{X_{1}}\;\;\;\;\;\;\;\;\;\;\;\;\\ X_{3}{=v}_{a}/K_{A}\;\;\;\;\\ X_{4}{=v}_{g}/K_{SSF}\;\\ X_{5}=\dot{X_{4}}\;\;\;\;\;\;\;\;\;\;\;\;\\ X_{6}{=v}_{out}/K_{P}\;\; \end{array}$$

(A14) $$\{\begin{array}{c} v_{set}{=K}_{EA}X_{1}\\ v_{a}{=K}_{A}X_{3}\\ v_{g}{=K}_{SSF}X_{4}\\ v_{out}{=K}_{P}X_{6} \end{array}$$
